# Hereditary angioedema: A national investigation of associated comorbidities and surgical procedures^☆^

**DOI:** 10.1016/j.waojou.2025.101136

**Published:** 2025-11-16

**Authors:** Lili Voloncs-Mindszenthy, Hanga Réka Horváth, Noémi Andrási, Tamás Szilágyi, Henriette Farkas

**Affiliations:** a Hungarian Angioedema Center of Reference and Excellence, Department of Internal Medicine and Haematology, Semmelweis University, Budapest, Hungary; b Doctoral School, Semmelweis University, Budapest, Hungary; c Pediatric Center, Tűzoltó Street Department, Semmelweis University, Budapest, Hungary; d Department of Orthopædics and Traumatology, St. John's Hospital, 1125, Budapest, Hungary

**Keywords:** Hereditary angioedema, C1-inhibitor deficiency, Comorbidity, Surgical procedures

## Abstract

**Background:**

Hereditary angioedema due to C1 inhibitor deficiency (HAE-C1INH) is characterized by sudden subcutaneous and/or submucosal angioedema attacks. C1 inhibitor is a serine protease inhibitor that regulates several enzyme cascade systems. The absence of this control raises concerns about the potential development of comorbidities.

**Objective:**

We aimed to investigate the comorbidities present in the Hungarian HAE-C1INH patient population and to examine the surgical procedures performed on these patients. Additionally, we sought to compare our results to those of the Hungarian general population.

**Methods:**

Demographical, clinical, laboratory, and radiographic data of all 178 adult HAE-C1INH patients followed up at the Hungarian Angioedema Center of Reference and Excellence were used to examine comorbidities and surgical procedures. Information about the general Hungarian population was extracted from national and European statistical databases and individual articles.

**Results:**

At least 1 comorbidity was present in 159 of our patients. From the observed 51 diseases, headache (58%), hypercholesterolemia (52%), hypertension (30%), and liver diseases (26%) were the most prevalent. Hypercholesterolemia and depression/anxiety were at least 3 times more common in the HAE-C1INH population as compared to the Hungarian general population. Tonsillectomy was performed 32, appendectomy 11, and inguinal hernioplasty 2 times more often before the diagnosis of HAE-C1INH was established. Every surgical procedure was more prevalent in the HAE-C1INH population.

**Conclusion:**

Regular, targeted screening is indispensable for the prevention and timely diagnosis of certain diseases found in higher prevalence in the HAE-C1INH population. The early identification and adequate treatment of angioedema attacks help prevent avoidable surgical interventions.

## Introduction

**Fig. 1:**
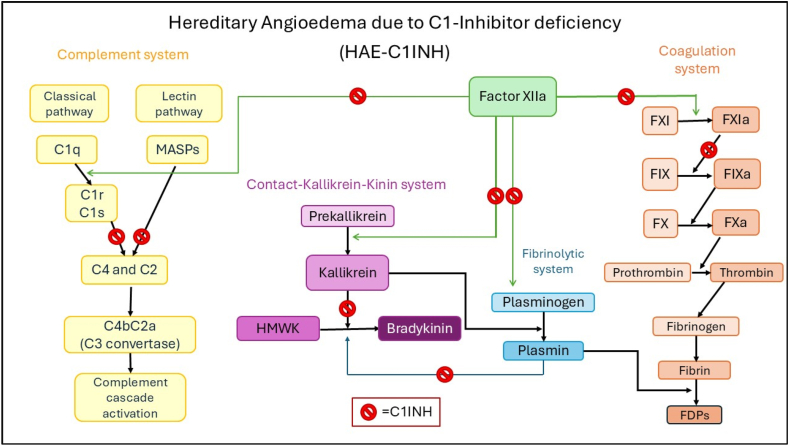
Pathomechanism of HAE. Pathomechanism of HAE: C1–INH plays a regulatory role in 4 enzyme cascade systems. When C1–INH is missing or dysfunctional, inhibition is released at the points indicated with the prohibitory sign. Abbreviations: C1INH=C1-inhibitor protein; FDPs = fibrin degradation products; HMWK = high molecular weight kininogen; MASPs = mannose-binding lectin (MBL)-associated serine protease.

The most common form of hereditary angioedema (HAE) is due to C1 inhibitor (C1INH) deficiency (HAE-C1INH).^1^ In this form, variants in the *SERPING1* gene result in decreased production of C1INH (type 1) or a dysfunctional protein (type 2). C1INH is a serine protease inhibitor that plays a critical regulatory role in the complement and coagulation cascades, along with the contact-kallikrein-kinin and fibrinolytic systems.^2^ The lack of C1INH leads to the activation of the contact-kallikrein-kinin system, which results in the cleavage of high molecular weight kininogen (HMWK) by plasma kallikrein, leading to an excessive bradykinin production. Bradykinin causes increased vascular permeability which manifests in the form of edema.^3^ Plasma kallikrein and factor XII play a role in the fibrinolytic system, where plasminogen is converted to plasmin, further contributing to the production of bradykinin by directly cleaving HMWK.^4^ In the coagulation cascade, factor XIIa causes a downstream activation of the coagulation system, leading to excessive thrombin activation.^5^ Thrombin further contributes to edema formation by increasing vascular permeability through the activation of protease activated receptors.^6^ In the complement cascade, C1INH induces the dissociation of C1r and C1s from C1q, which keeps the rest of the cascade in check (Fig. 1).^7^ In the absence of regulation by C1INH, the fine balance of these systems is disrupted, potentially predisposing HAE patients for comorbidities. For example, consumption of C4 and C3 complements by uncontrolled cleavage can predispose patients to autoimmune diseases.^8^

The clinical manifestation of HAE-C1INH is characterized by unpredictable subcutaneous and/or submucosal edema formation. The appearance of an edema attack is not only an exasperating experience for patients but also a situation that oftentimes warrants medical attention. Therefore, clinical characterization of HAE patients is important as it facilitates accurate recognition and management by treating physicians. As discussed above, the pathomechanism of HAE-C1INH might predispose patients to the development of various comorbidities.^2^^,^^9^, ^10^, ^11^, ^12^, ^13^, ^14^, ^15^ Conversely, these associated conditions may also impact the course and severity of HAE.^16^

We addressed these matters by conducting a large-scale longitudinal study, investigating comorbidities and surgical procedures that occurred in our adult HAE-C1INH population. The first objective of our study was to describe the prevalence of various comorbidities in the adult HAE-C1INH population. Subsequently, we aimed to examine the differences between our HAE-C1INH patient population and the general Hungarian population in terms of comorbidities. Our third aim was to determine the specific types of surgical procedures that the Hungarian HAE-C1INH population underwent at any point throughout their lives before and after their HAE-C1INH diagnosis. Finally, we compared the prevalence of these surgical procedures to the non-HAE adult patient population.

## Methods

### Patients

We conducted a longitudinal study with both prospective and retrospective components on 178 Hungarian adult HAE-C1INH patients followed at the Hungarian Angioedema Center of Reference and Excellence between 1979 and 2023. Data on comorbidities were collected prospectively through a predefined registry protocol, while information on surgical history before diagnosis and control population data were obtained retrospectively. Diagnosis of HAE-C1INH was made based on clinical symptoms, family history, complement studies (C1q, C4, C1INH antigenic concentration, C1INH functional assessment), and genetic studies (disease-causing variants in the *SERPING1* gene) in every patient. In both analyses, we included patients who were at least 18 years of age at the time of database closure in November 2023. Data on patients were stratified into 7 age groups (18–25, 25–35, 35–45, 45–55, 55–65, 65–75, and 75+years). The age groups were created based on the datasets of our control sources: The Statistical Authority of the European Union (EUROSTAT)^17^ and the Hungarian Central Statistical Office (HCSO).^18^

### Hungarian HAE registry

The Hungarian Angioedema Center of Reference and Excellence established a comprehensive registry in 1979 with the aim of collecting and documenting detailed medical information on each Hungarian HAE patient. Demographic, clinical, laboratory, and radiographic data, along with all medical reports from any healthcare institution, are collected. Upon being diagnosed with HAE at our clinic, medical reports about any previously diagnosed conditions and surgical procedures are gathered from the patients and recorded into our registry. Once registered, patients come for follow-ups every 3 months during the first year after their HAE diagnosis and then annually afterwards. Any changes in laboratory values, newly diagnosed diseases, surgical procedures, and a detailed account of the patients’ HAE-related symptoms are noted each year. The Registry operates under approval from the Hungarian Medical Research Council (license number: 19068-5/2018/EKU) and the study was conducted in accordance with the Declaration of Helsinki.

### Comorbidities

To investigate comorbidities, we searched our patients’ complete medical history from the time they turned 18 years old or the time they were diagnosed with HAE (whichever was later) using all available documentation about the patients in the HAE Registry. Using the latest 11th revision of the International Classification of Diseases (ICD11),^19^ we systematized the 51 observed conditions into 11 classes (Supplementary Table 3). In ICD11, diseases are grouped mainly according to organ systems, out of which we looked at the digestive, musculoskeletal, endocrine, respiratory, circulatory, genitourinary, nervous and the immune system. Other classes included neoplasms, mental, behavioral, and neurodevelopmental diseases, and sleep-wake disorders.^19^ In order to gain information about the general Hungarian population, we used the databases of EUROSTAT and HCSO, along with any relevant publications on specific conditions (Table 1). For each disease in each age group, we calculated the prevalence and the 95% confidence interval (CI, calculated using a normal approximation) of the prevalence of the disease in the HAE-C1INH population.

**Table 1:** Sources of information for diseases in the general Hungarian population.

EUROSTAT^17^	HCSO^18^	Individual publications
• Spondylosis (degenerative conditions of the spine) • Arthrosis (osteoarthritis of wrist, hand, hip, knee, or unspecified) • Diabetes • Asthma • COPD • Depression • Allergies • Hypercholesterolemia • Coronary artery disease • Myocardial infarction • Hypertension • Stroke • Kidney diseases	• Neoplasms • Osteoporosis • Thyroid diseases	• GERD^54^ • *Helicobacter pylori* seropositivity^55^ • Lactose intolerance^56^ • Celiac disease^57^ • Hemorrhoids^58^ • IBD^59^ • Heart failure^60^ • BPH^61^ • PCOS^62^ • Epilepsy^63^ • Insomnia^64^ • Eczema^65^

Abbreviations: BPH=Benign prostatic hyperplasia; COPD=Chronic obstructive pulmonary disease; EUROSTAT = European Statistics; Statistical Authority of the European Union, GERD = gastroesophageal reflux disease; HCSO = Hungarian Central Statistical Office; IBD= Inflammatory bowel disease; PCOS= Polycystic ovary syndrome

### Surgical procedures

For the examination of surgical procedures, we observed each patient's medical history before and after their HAE diagnosis, so that these analyses encompassed their entire lifetime. To ensure consistency in the sources of information for both comorbidities and surgical procedures, we utilized the *Surgical Operations and Procedures Report* from EUROSTAT.^20^ Analyzed surgical interventions included appendectomy, cholecystectomy, inguinal hernioplasty, tonsillectomy and tracheotomy. We expressed the prevalence of each surgical procedure for every 1000 patient-years for HAE-C1INH patients before and after the diagnosis, as well as for the overall HAE-C1INH and the overall Hungarian population.

### Statistical analysis

For each disease in each age group, we calculated the ratio of the prevalence (in percentage) in the HAE-C1INH population and the prevalence (in percentage) in the Hungarian population. To determine whether the observed differences were statistically significant, we also compared the data of the Hungarian general population against the HAE-C1INH population's CI. Mathematical significance was established when the Hungarian general population's data fell outside of the 95% CI.

### Specific subgroup analyses

In order to check for an association between danazol taken as a long-term prophylaxis (LTP) and hyperlipidemia, we performed a Chi square analysis for each age group separately. Similarly, we performed Fisher's exact test to check for an association between danazol LTP and osteoporosis. Statistical significance was set to 0.05.

In order to assess the effect that age, sex, and body mass index (BMI) have on hyperlipidemia, we performed a multiple logistic regression analysis on data from a randomly selected year from every patient. BMI categories were defined according to the World Health Organisation (WHO) classification system.^21^ Statistical significance was set to 0.05.

To address the temporal fluctuation of psychological conditions, like depression and anxiety, we examined the prevalence of depression in our cohort at 2 specific time points (2014 and 2019), selected based on the availability of population-level data from EUROSTAT. We cross-referenced patient documentation for those years to assess point prevalence.

All calculations were made using Microsoft Excel and GraphPad Prism 9 programs.

## Results

### Patient demographics

Our study included the entire diagnosed adult Hungarian HAE-C1INH population of 178 patients from 74 families. Out of the 178 patients, 95 were female and 83 were male. 173 patients were diagnosed with HAE-C1INH type 1 and 5 patients with HAE-C1INH type 2. Their complete demographic data can be viewed in Table 2. The median age at database closure (Nov. 30, 2023) was 44.63 years (IQR 33.97–57.58) and the median age at diagnosis was 27.15 years (IQR 13.74–41.55). The types of LTPs along with risk factors in different age groups are summarized in Table 3. Seventy-two (40.45%) patients were taking danazol, 26 (14.6%) were taking tranexamic acid, and 12 (6.74%) were taking C1INH concentrate as LTP during the observation period. Of the 12 patients on intravenous (IV) C1 inhibitor concentrate, 11 were treated with IV plasma-derived C1INH. One patient received off-label IV recombinant human C1INH for 3 months due to temporary limited access to IV plasma derived C1INH concentrate for LTP. Lanadelumab and subcutaneous plasma-derived C1–INH (sc-pd-C1INH) became available in Hungary only from 2022 onwards, while berotralstat was not available during the study period at all. Due to the minimal follow-up time with lanadelumab and sc-pd-C1INH, along with the unavailability of berotralstat, these therapies were not included in our analysis. Furthermore, during the short observation period, no new concomitant disorders were observed in our cohort.

**Table 2:** Demographical data of the HAE-C1INH patient population.

Demographic data of the HAE population	
Total number of patients (n)	178
HAE-C1INH type I (n)	173
HAE-C1INH type II (n)	5
Number of males	95 (53.4%)
Number of females	83 (46.6%)
Average age at data closure (years)	49 (min: 18, max: 93)
Average age at diagnosis (years)	26 (min: 1, max: 77)

Abbreviations: HAE-C1INH=Hereditary angioedema due to C1INH deficiency

**Table 3:** LTP and risk factors in the HAE-C1INH patient population.

Age groups		Number of patients	Long term prophylaxis and risk factors					
Start	End		BMI	Danazol prophylaxis	TXA prophylaxis	C1INH prophylaxis	Smoke	Alcohol
18	25	93	23.26	20	9	3	12	14
25	35	105	25.16	33	11	5	19	24
35	45	103	27.56	40	10	2	15	20
45	55	79	27.51	38	4	3	16	21
55	65	56	28.05	25	2	1	11	14
65	75	27	27.16	10	1	0	4	8
75		10	25.02	3	0	0	0	1
Total:		178	Total:	72	26	12	49	71

Abbreviations: BMI=Body mass index, LTP = Long-term prophylaxis, TXA = tranexamic acid

### Comorbidities: Hungarian HAE-C1INH population based on age groups

Here, we highlight 2 comorbidities that stood out in our cohort due to their distinctive characteristics.

Liver abnormalities were present in 25.84% of our cohort. Most commonly hepatic steatosis with or without associated hepatomegaly; less frequently, hepatic cysts and hemangiomas were observed. No cases of viral hepatitis or other clinically significant chronic liver pathologies were identified. Headaches emerged as the most frequently reported comorbidity in our patient population with a prevalence of 57.87% and similarly high rates across all age groups (Supplementary Table 1).

### Comorbidities: HAE-C1INH population versus the general population based on age groups

Among the cardiovascular diseases, only hypercholesterolemia demonstrated a significant difference between the HAE-C1INH and the general population as well as across all the age groups. While no significant difference was found for hypertension at the population level, we observed notable differences in the second and third age groups. The other cardiovascular conditions only presented significant differences on individual age group levels. Danazol LTP did not have a significant effect on hypercholesterolemia (p > 0.05 for all age groups). On the other hand, age and sex were significant predictors of hypercholesterolemia in our cohort, while BMI was not statistically significant (Fig. 4).

Regarding depression and anxiety, the HAE-C1INH population was 3.37 times more affected than the general population. In the 2nd, 3rd and 5th age groups, HAE-C1INH patients were 2.43, 3.24, and 4.09 times more affected respectively. In 2014, 9 of 150 patients (6.0%) had documented depression, compared to 4.9% in the Hungarian general population. In 2019, 16 of 168 patients (9.5%) were affected, versus 4.0% in the general population. In our cohort, 14 out of 24 patients received androgens at some point during their treatment. Some patients developed psychiatric disorders prior to the initiation of androgen therapy, whereas others developed these conditions in the later years of treatment or after discontinuing the medications. Therefore, proper statistical analysis could not be performed to investigate the association of androgen usage and psychiatric disorders.

Spondylosis affected only 11.79% of the HAE-C1INH population, while 31.3% of the general population. In addition, for this disease, a statistically significant difference was observed in every age group with the general population having an at least 2.3 times higher prevalence. Arthrosis affected 12.9% of the HAE-C1INH population and 18.0% of the general population. A significantly lower proportion of HAE-C1INH patients were affected compared to the Hungarian population in the 5th, 6th, and 7th age groups. Osteoporosis affected a 2.8 times smaller proportion of the HAE-C1INH population as compared to the general population. For this disease, significant difference was observed only in the 6th age group. Danazol LTP did not have a significant effect on osteoporosis (p > 0.05 for all age groups).

Regarding the documented allergies, there was no substantial difference between the 2 populations, while a major discrepancy was observed in the 7th age group. No divergence was observed between the HAE-C1INH and the general Hungarian populations in the prevalence of neoplasms. However, the first and second HAE-C1INH age groups represented a 5.37 and a 1.90-fold increase in prevalence compared to the age groups of the Hungarian general population, respectively, which were statistically not significant. The prevalence of diabetes, thyroid disorders, asthma, and COPD were without major deviations from the general population (Table 4). The prevalence of each disease found in the HAE-C1INH population is detailed in Table 1 of the Supplemental Appendix.

**Table 4:**
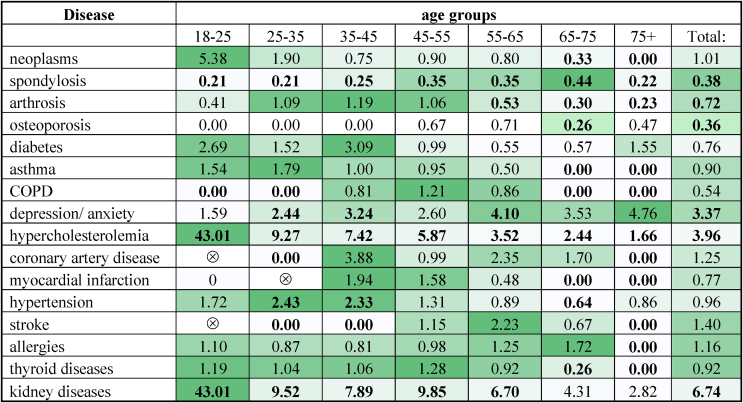
Prevalence of diseases in the HAE-C1INH patient population versus the Hungarian general population expressed in ratios (HAE-C1INH patient population/Hungarian general population)

The color scale indicates the relative size of the ratios. Darker colors indicate higher values. Bolded values indicate statistical significance. The “⊗” sign indicates a zero value in the Hungarian general population, resulting in an uninterpretable quotient.

Abbreviations: COPD=Chronic obstructive pulmonary disease

### Comorbidities: HAE-C1INH population versus the general population as a whole

We also compared specific diseases between the 2 populations for which data was only available for the entire Hungarian population. When comparing the 2 populations, we observed a significant difference in lactose intolerance, hemorrhoids, insomnia, and eczema (ratios: 0.12, 0.45, 0.37, and 0.34, respectively) (Table 2 in the Supplemental Appendix).

### Surgical procedures

#### Surgical procedures in the HAE-C1INH population before and after their HAE diagnosis

**Fig. 2:**
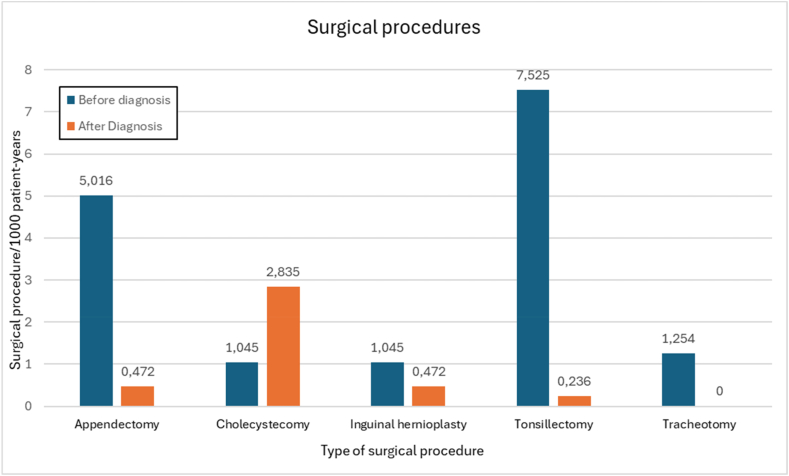
Prevalence of surgical procedures before and after the diagnosis of HAE. The prevalence of 5 surgical procedures expressed in number of procedures per 1000 patient-years. Some patients may have undergone more than 1 surgical procedure

Tonsillectomies and appendectomies were the 2 surgical procedures that were performed substantially more often before than after the diagnosis of HAE-C1INH: 31.88 and 10.62 times more, respectively. Out of the 37 tonsillectomies, only 1 was performed post-diagnosis in an adult patient and another 1 was performed after the year 2000. All other tonsillectomies happened before the year 2000, before the diagnosis of HAE-C1INH. Regarding the age distribution of tonsillectomies, 72.97% (27/37) of our patients were under 18 years of age and the rest of the patients were adults. Notably, tracheostomies were performed exclusively before the diagnosis of HAE-C1INH was established, involving 6 patients. In the case of inguinal hernioplasty, 2.21 times more patients were operated before diagnosis. Cholecystectomy was the only surgical operation that was done more frequently after diagnosis. It was performed 2.71 times more following the diagnosis than before. Out of the 17 patients who underwent cholecystectomy, 64.70% (11/17) of patients had concurrent hypercholesterolemia, 64.70% (11/17) were taking danazol as LTP and both were true for 41.17% (7/17) of the patients. 70.58% (12/17) of the patients were either in or above the 3rd age group (Fig. 2).

#### Surgical procedures in the HAE-C1INH population vs the general Hungarian population

**Fig. 3:**
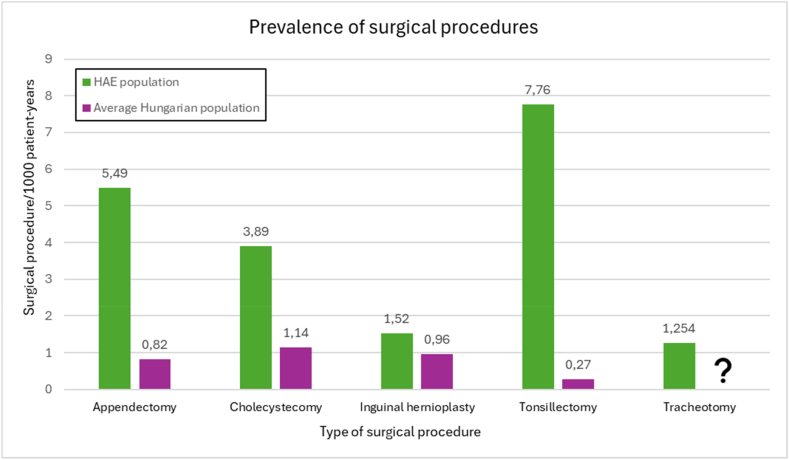
Prevalence of surgical procedures in the HAE patient population versus the Hungarian general population. The prevalence of 5 surgical procedures expressed in number of procedures per 1000 patient-years for the HAE patient population and the Hungarian general population. The question mark indicates the lack of statistical information regarding this surgical procedure in the Hungarian general population

Every operation was more prevalent in the HAE-C1INH population. Tonsillectomies and appendectomies were 28.74 and 6.69 times more common, while cholecystectomies and inguinal hernioplasties were 3.41 and 1.58 times more common in the HAE-C1INH population. There was no data available regarding tracheostomy operations in the Hungarian general population (Fig. 3).

**Fig. 4:**
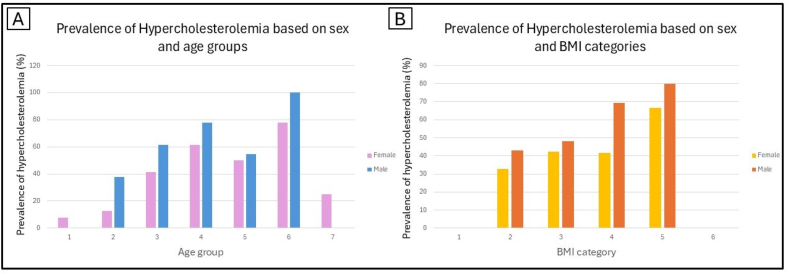
Prevalence of Hypercholesterolemia based on sex and age groups (A), and sex and BMI categories (B). Age group numbers (1–7) correspond to the respective age group categories: 18–25, 25–35, 35–45, 45–55, 55–65, 65–75, and 75+years. BMI categories (1–6) correspond to the respective BMI classes: 1 = BMI <18.5 kg/m^2^, 2 = BMI between 18.5 and 24.9 kg/m^2^, 3 = BMI between 25 and 29.9 kg/m^2^, 4 = BMI between 30 and 34.9 kg/m^2^, 5 = BMI between 35 and 39.9 kg/m^2^, 6 = BMI of 40 kg/m^2^ or higher. Abbreviations: BMI = body mass index

## Discussion

In our large-scale longitudinal study assessing 51 diseases and 5 surgical procedures in Hungarian HAE-C1INH patients throughout their lifetime—categorized into 7 age groups—we observed an increased risk of hyperlipidemia and a higher prevalence of surgical procedures in HAE-C11INH patients as compared to the general Hungarian population. To the best of our knowledge, 4 major national studies focused specifically on the association between comorbidities and HAE: Björkmann et al,^10^ Zanichelli et al,^11^ Steiner et al,^15^ and Christiansen et al.^12^ It is important to note that the methods of data collection and processing varied across the studies.

At least 1 comorbidity was present in 89.32% of our patients. We found a great proportion (25.84%) of patients with liver disorders. Hypercholesterolemia was also present in 81.08% of our patients with hepatic steatosis, probably explaining the high number of liver disorders, as the association between elevated cholesterol levels and hepatic steatosis is well-established.^22^ Jiang et al^23^ recently published that certain *SERPING1* mutations lead to C1INH aggregation within the endoplasmic reticulum, leading to cell apoptosis. Given that C1INH is primarily produced in the liver,^24^ this mechanism could potentially lead to diffuse hepatic injury.

Analysis of headaches is a quite difficult task, as symptom assessment is usually based on subjective experience.^25^ However, in 2 of our patients, we observed a unique type of headache that might have been cerebral HAE attack episodes as they resolved with C1INH administration but not analgesics. As described before, the local compressive effect of a cerebral edema may produce various neurological signs and non-specific pain experienced as headaches.^26^

Our findings revealed a 3.95-fold increase in hypercholesterolemia as compared to the general population, independently of danazol LTP. Additionally, multiple reports have documented that danazol used in the lowest effective dose does not impact liver function.^27^ However, ours is the first report on the elevation of cholesterol levels in the HAE-C1INH population independently of the usage of danazol therapy. The cause of the elevated LDL can be multifactorial. While data on specific dietary and physical activity patterns aren't available in our cohort, it is well established that avoidance of certain food groups and the reduction of physical activity strongly influence cholesterol levels^28^^,^^29^ HAE patients of our cohort have reported that their attacks are triggered by physical exercise and certain food groups, causing them to lead an inactive lifestyle and avoiding such food groups.^30^ It is also known that prekallikrein levels are associated with hypercholesterolemia,^31^ although the specific significance of this relationship in HAE remains to be investigated.

We observed a higher prevalence of hypertension in the second and third age groups within the HAE-C1INH population compared to the general population. The earlier onset of hypertension in our patients can be attributed to the earlier exposure to close medical observation as part of the follow-up by the HAE center, as reported by Björkman et al^10^ In addition to the increased medical surveillance our patients undergo, it is important to highlight that the underlying endothelial dysfunction characteristic of HAE-C1INH, seen both during and between attacks, may contribute directly to the earlier onset of vascular pathologies such as hypertension.^32^, ^33^, ^34^

The rate of depression and anxiety was significantly higher in the Hungarian HAE-C1INH population than in the general population, as could be expected from the unpredictability of the disease. This is a well-documented phenomenon in HAE patients.^35^^,^^36^ In our patient population, psychiatric comorbidities were present in some patients both before and after androgen exposure, with onset and course fluctuating over time, suggesting that while androgens may contribute, these conditions likely reflect multifactorial influences beyond therapy alone.

Arthrosis and osteoporosis both had lower prevalence in the HAE-C1INH population, than in the general population. An analysis on the US HAE population has shown similar trends.^12^ The seemingly protective effect of HAE-C1INH might be partially attributed to danazol LTP. Taking the lowest effective dose of danazol did not cause osteoporosis and may have even protected Hungarian HAE patients from mineral loss.^37^ The unexpectedly lower prevalence of spondylosis in all HAE-C1INH groups may be due to patients not spontaneously disclosing this condition unless specifically asked. In contrast, the control group was directly asked whether they had spondylosis.^38^

Laboratory-confirmed diagnoses may not always correlate with the clinical presentation of allergic diseases, rendering the assessment of the condition more difficult.^39^ The prevalence of self-reported allergy in the general Hungarian population is 19.3%.^17^ In a previous study of our group, patient-reported hypersensitivity symptoms were present in 63.2% of the Hungarian HAE-C1INH population.^40^ In our current study, we examined laboratory parameters, including total IgE levels and allergy-specific antigen tests, to confirm every self-reported allergy. We observed that 22.47% of our HAE-C1INH population had allergic diseases. Consistent with our earlier study, we didn't observe any significant differences in the prevalence of autoimmune diseases when comparing the HAE-C1INH and the general Hungarian population.^41^ The association between autoimmune disorders and HAE-C1INH is well-documented; however, studies investigating this connection report widely differing prevalence rates.^8^, ^9^, ^10^

No difference was observed in the prevalence of neoplasms when comparing our HAE-C1INH population to the general population. This is in line with the observation of Björkman et al in the Swedish population.^10^ The prevalence of diabetes, thyroid disorders, asthma, and COPD in the HAE-C1INH population were like that of the general population as a whole. Zanichelli et al reported similar results for diabetes and thyroid diseases.^11^ Christiansen et al reported similar results for asthma, COPD, thyroid disorders and diabetes. Steiner et al didn't analyze the incidence of these diseases.^15^ Regarding deep vein thrombosis and pulmonary embolism, we were unable to find comparable data for the Hungarian general population. This is primarily due to a substantial proportion of cases remaining unidentified because of their asymptomatic nature.^42^

To the best of our knowledge, the prevalence of tonsillectomy in HAE populations has not yet been investigated. The indications for performing a tonsillectomy have been refined over the last 4 decades, with fewer conditions warranting the procedure. This paradigm shift was facilitated by the emergence of the Paradise criteria in 1984.^43^ In our HAE-C1INH population, 72.97% of all tonsillectomies occurred in childhood, most of them before the above-mentioned changes were implemented in Hungary. Therefore, a number of these cases could have been unnecessary, accounting for a greater number of procedures in that era. Additionally, recurrent tonsillitis is more common in children, increasing the likelihood of a tonsillectomy being performed.^44^ Moreover, pharyngeal edema attacks can mimic recurrent tonsillitis, leading to surgeries based on a misdiagnosis, further explaining the increased prevalence of tonsillectomies before the diagnosis of HAE-C1INH. We do not have enough histological reports to determine how many of the surgeries performed in our HAE-C1INH population were required.

The increased prevalence of abdominal exploratory surgeries in HAE-C1INH patients, specifically appendectomies, has been documented by several research teams over the years.^11^^,^^45^^,^^46^ In fact, Zanichelli et al found that the second most common misdiagnosis in HAE-C1INH patients was appendicitis after allergic angioedema.^46^ Kayikci et al also reported recently that abdominal surgeries were the second most common surgical procedure overall and the most frequent prior to the diagnosis of HAE.^47^ Our findings in the Hungarian HAE-C1INH population further support this observation. Furthermore, appendectomies are also more commonly performed in the pediatric population, due to the younger age of onset of appendicitis.^48^^,^^49^ Abdominal HAE-C1INH attacks can mimic an acute abdomen, prompting doctors to perform an immediate operation without thoroughly considering differential diagnosis. The emergency room approach is justifiable; however, the incorporation of a more thorough anamnesis would be advisable to lower negative appendectomy rates.^50^ Inguinal hernioplasty procedures were performed 2.21 times more frequently before the diagnosis of HAE, as a lower abdominal or subcutaneous edema attack is also hypothesized to mimic an inguinal hernia.

Tracheostomies were performed only before our patients’ HAE diagnosis. In the absence of a HAE-C1INH diagnosis, patients experiencing a sudden laryngeal edema attack are treated with this life-saving procedure. Once the correct diagnosis is established, the appropriate on-demand therapy can be utilized to immediately alleviate the suffocating attack. As previously published in a paper on the Hungarian HAE-C1INH population, 13 patients, who were undiagnosed at the time, died in a laryngeal attack. Two diagnosed HAE-C1INH patients died of laryngeal edema due to non-compliance.^51^ No more deaths from laryngeal attacks were documented in our population since the time of that publication.

Only cholecystectomy was performed more often after diagnosis. According to a large meta-analysis recently published by Wang et al, cholelithiasis develops with advancing age, with the greatest incidence occurring in individuals over 70 years old.^52^ Since the average age at which diagnosis was established in our patients is 26 years, patients were more likely to develop cholelithiasis and subsequent cholecystectomy after the diagnosis.

When comparing the Hungarian HAE-C1INH population and the Hungarian general population in terms of surgical interventions, we observed an increased prevalence of every procedure in the HAE-C1INH population. The reason for this difference is primarily attributable to the factors mentioned earlier in the case of appendectomy, tonsillectomy, and inguinal hernioplasty. However, in the case of cholecystectomy, it is important to point out an additional HAE-related feature that influences this difference between the 2 populations. 64.70% of our patients who underwent cholecystectomy had concurrent hypercholesterolemia. The direct relationship between serum LDL cholesterol and the development of cholesterol gallstones has been well established in the literature.^53^ Therefore, since hypercholesterolemia is more prevalent in our HAE-C1INH population, a higher rate of cholelithiasis (and subsequent cholecystectomy) can also be expected.

Our study has its limitations. Firstly, the number of patients is quite low in the older age groups. As a result, percentage distribution does not properly describe these groups, skewing the results of the comparison with the general population. Over time, as our patients age, we will be able to correct this discrepancy. Secondly, differences in data collection methods across the sources must also be considered. EUROSTAT and HCSO used questionnaires to obtain information, while we used clinical data confirmed by doctors. Although registry data are entered by healthcare professionals based on medical records, we acknowledge the potential for documentation-related bias, as the completeness of prior records may affect the accuracy of retrospective comorbidity data. Regarding the statistical methodology, we acknowledge that using percentage-based comparisons is less robust than reporting odds ratios or relative risks. However, since our control sources did not disclose their raw data (total sample size or event counts), the calculation of such metrics were deemed impossible. Reporting percentage-based comparisons were the most accurate and feasible method under these circumstances. Finally, our data encompasses the patients' entire lifetimes, rather than being limited to information gathered within a single year, as seen in the control sources. Limitations of a study can raise awareness of the weaknesses in a particular field. Our large, longitudinal study that encompasses a wide range of diseases highlights the necessity for standardized data collection and reporting.

To conclude, our analysis showed that HAE-C1INH patients seem to have an increased risk of hypercholesterolemia and a decreased risk of osteoporosis than the general population. No serious, life-threatening comorbidities were more common in the HAE-C1INH population, and laryngeal edema could effectively be managed after the diagnosis of HAE, enabling physicians to reassure HAE-C1INH patients about the impact of their disease on their lives.

## Abbreviations

BMI, Body Mass Index; C1INH, C1 inhibitor protein; CI, Confidence interval; EUROSTAT, European Statistics; Statistical Authority of the European Union; HAE, Hereditary angioedema; HAE-C1INH, Hereditary angioedema due to C1 inhibitor deficiency; HCSO, Hungarian Central Statistical Office; HMWK, High molecular weight kininogen; ICD11, 11th International Classification of Diseases; IV, Intravenous; LTP, Long-term prophylaxis; sc-pd-C1INH, subcutaneous plasma-derived C1INH.

## Availability of data and materials

The datasets generated during and/or analyzed during the current study are available from the corresponding author on reasonable request.

## Author contributions

Concept and design: Lili VOLONCS-MINDSZENTHY, Hanga Réka HORVÁTH, Henriette FARKAS.

Collection of data: Lili VOLONCS-MINDSZENTHY, Noémi ANDRÁSI, Tamás SZILÁGYI.

Data analysis: Lili VOLONCS-MINDSZENTHY, Hanga Réka HORVÁTH, Henriette FARKAS.

Manuscript writing: Lili VOLONCS-MINDSZENTHY, Hanga Réka HORVÁTH, Henriette FARKAS.

Final approval of manuscript: All authors.

## Ethics statement

The study protocol was approved by the institutional review board of Semmelweis University, Budapest, and informed consent was obtained from the participants in accordance with the Declaration of Helsinki. The Hungarian HAE Registry operates under approval from the Hungarian Medical Research Council (license number: 19068-5/2018/EKU).

## Authors’ consent for publication

All authors give their consent to the publication of this work.

## Submission declaration

The authors hereby declare that this manuscript has not been published previously (except in the form of an abstract and academic thesis), that it is not under consideration for publication elsewhere, that its publication is approved by all authors and tacitly by the responsible authorities, and that, if accepted, it will not be published elsewhere in the same form, in English or in any other language, including electronically without the written consent of the copyright holder.

## Declaration of Generative AI and AI-assisted technologies in the writing process

Nothing to disclose.

## Funding

This study received no funding nor specific grants from any sector.

## Declaration of competing interest

Lili Voloncs-Mindszenthy has no conflict of interests to declare.

Hanga Réka Horváth received travel grants from Takeda and CSL Behring.

Noémi Andrási has no conflict of interest to declare.

Tamás Szilágyi has no conflict of interest to declare.

Henriette Farkas has received research grants from CSL Behring, Takeda and Pharming and served as an advisor for these companies and Kalvista, Intellia, Ionis, Pharvaris, ONO Pharmaceuticals and Biocryst and has participated in clinical trials/registries for BioCryst, CSL Behring, Pharming, Kalvista, Pharvaris and Takeda.

## Footnotes

Appendix A Supplementary data to this article can be found online at https://doi.org/10.1016/j.waojou.2025.101136.
