# Supplementary material for: Hereditary angioedema: A national investigation of associated comorbidities and surgical procedures
Source: World Allergy Organ J. 2025 Nov 16;18(11):101136. doi: 10.1016/j.waojou.2025.101136 (PMC12664378; doi:10.1016/j.waojou.2025.101136)
Supplement: Multimedia component 1 [file mmc1.docx]

|  | **Age** **groups** | | | | | | | |
| --- | --- | --- | --- | --- | --- | --- | --- | --- |
| **Diseases (n, percentage)** | 18-25 | 25-35 | 35-45 | 45-55 | 55-65 | 65-75 | 75+ | Total: |
| Number of patients | 93 | 105 | 103 | 79 | 56 | 27 | 10 | 178 |
| GERD | 8, 8.60 | 12, 11.43 | 13, 12.62 | 22, 27.85 | 14, 25.00 | 7, 25.93 | 1, 10.00 | 37, 20.79 |
| *Helicobacter pylori* infection | 3, 3.23 | 5, 4.76 | 9, 8.74 | 10, 12.66 | 5, 8.93 | 2, 7.41 | 0, 0.00 | 31, 17.42 |
| Lactose intolerance | 5, 5.38 | 2, 1.90 | 4, 3.88 | 1, 1.27 | 0, 0.00 | 0, 0.00 | 0, 0.00 | 8, 4.49 |
| Cholecystolithiasis | 5, 5.38 | 4, 3.81 | 3, 2.91 | 8, 10.13 | 7, 12.50 | 3, 11.11 | 0, 0.00 | 19, 10.67 |
| Stomach ulcer | 0, 0.00 | 0, 0.00 | 0, 0.00 | 1, 1.27 | 3, 5.36 | 1, 3.70 | 0, 0.00 | 3, 1.69 |
| Intussusception | 0, 0.00 | 0, 0.00 | 0, 0.00 | 0, 0.00 | 0, 0.00 | 0, 0.00 | 0, 0.00 | 0, 0.00 |
| Celiac disease | 4, 4.30 | 3, 2.86 | 2, 1.94 | 0, 0.00 | 0, 0.00 | 0, 0.00 | 0, 0.00 | 5, 2.81 |
| Polyp of gallbladder | 0, 0.00 | 4, 3.81 | 9, 8.74 | 8, 10.13 | 4, 7.14 | 0, 0.00 | 0, 0.00 | 12, 6.74 |
| Hemorrhoids | 0, 0.00 | 2, 1.90 | 3, 2.91 | 2, 2.53 | 3, 5.36 | 2, 7.41 | 1, 10.00 | 8, 4.49 |
| Liver disorders | 7, 7.53 | 15, 14.29 | 23, 22.33 | 25, 31.65 | 17, 30.36 | 5, 18.52 | 1, 10.00 | 46, 25.84 |
| IBD | 1, 1.08 | 1, 0.95 | 2, 1.94 | 1, 1.27 | 1, 1.79 | 0, 0.00 | 0, 0.00 | 2, 1.12 |
| Acute pancreatitis | 1, 1.08 | 0, 0.00 | 0, 0.00 | 1, 1.27 | 1, 1.79 | 0, 0.00 | 0, 0.00 | 3, 1.69 |
| Neoplasms | 1, 1.08 | 1, 0.95 | 1, 0.97 | 2, 2.53 | 3, 5.36 | 1, 3.70 | 0, 0.00 | 9, 5.06 |
| Spondylosis | 2, 2.15 | 5, 4.76 | 7, 6.80 | 9, 11.39 | 8, 14.29 | 5, 18.52 | 1, 10.00 | 21, 11.80 |
| Arthrosis | 1, 1.08 | 4, 3.81 | 8, 7.77 | 14, 17.72 | 8, 14.29 | 3, 11.11 | 1, 10.00 | 23, 12.92 |
| Osteoporosis | 0, 0.00 | 0, 0.00 | 0, 0.00 | 1, 1.27 | 3, 5.36 | 1, 3.70 | 1, 10.00 | 4, 2.25 |
| Diabetes | 2, 2.15 | 4, 3.81 | 7, 6.80 | 5, 6.33 | 5, 8.93 | 3, 11.11 | 3, 30.00 | 12, 6.74 |
| Hypothyroidism | 2, 2.15 | 2, 1.90 | 4, 3.88 | 5, 6.33 | 3, 5.36 | 0, 0.00 | 0, 0.00 | 10, 5.62 |
| Goiter | 0, 0.00 | 1, 0.95 | 2, 1.94 | 2, 2.53 | 1, 1.79 | 0, 0.00 | 0, 0.00 | 3, 1.69 |
| Thyroiditis | 3, 3.23 | 3, 2.86 | 5, 4.85 | 3, 3.80 | 3, 5.36 | 1, 3.70 | 0, 0.00 | 9, 5.06 |
| Hyperthyroidism | 0, 0.00 | 0, 0.00 | 0, 0.00 | 1, 1.27 | 1, 1.79 | 0, 0.00 | 0, 0.00 | 1, 0.56 |
| COPD | 0, 0.00 | 0, 0.00 | 2, 1.94 | 4, 5.06 | 3, 5.36 | 0, 0.00 | 0, 0.00 | 4, 2.25 |
| PTX | 0, 0.00 | 0, 0.00 | 2, 1.94 | 0, 0.00 | 0, 0.00 | 0, 0.00 | 0, 0.00 | 2, 1.12 |
| Asthma bronchiale | 5, 5.38 | 6, 5.71 | 4, 3.88 | 3, 3.80 | 2, 3.57 | 0, 0.00 | 0, 0.00 | 8, 4.49 |
| Anxiety/depression | 4, 4.30 | 11, 10.48 | 10, 9.71 | 8, 10.13 | 14, 25.00 | 4, 14.81 | 2, 20.00 | 24, 13.48 |
| Hyperlipidaemia | 7, 7.53 | 13, 12.38 | 18, 17.48 | 20, 25.32 | 19, 33.93 | 6, 22.22 | 0, 0.00 | 42, 23.60 |
| Hypercholesterinaemia | 16, 17.20 | 37, 35.24 | 52, 50.49 | 58, 73.42 | 43, 76.79 | 18, 66.67 | 4, 40.00 | 93, 52.25 |
| Coronary artery disease | 1, 1.08 | 0, 0.00 | 2, 1.94 | 5, 6.33 | 5, 8.93 | 4, 14.81 | 0, 0.00 | 8, 4.49 |
| Myocardial infarction | 0, 0.00 | 0, 0.00 | 1, 0.97 | 1, 1.27 | 1, 1.79 | 0, 0.00 | 0, 0.00 | 3, 1.69 |
| Hypertension | 4, 4.30 | 13, 12.38 | 30, 29.13 | 31, 39.24 | 26, 46.43 | 11, 40.74 | 6, 60.00 | 54, 30.34 |
| Heart failure | 0, 0.00 | 1, 0.95 | 1, 0.97 | 5, 6.33 | 3, 5.36 | 2, 7.41 | 1, 10.00 | 8, 4.49 |
| Thrombosis | 2, 2.15 | 1, 0.95 | 0, 0.00 | 1, 1.27 | 1, 1.79 | 1, 3.70 | 0, 0.00 | 6, 3.37 |
| Stroke | 0, 0.00 | 0, 0.00 | 0, 0.00 | 1, 1.27 | 3, 5.36 | 1, 3.70 | 0, 0.00 | 5, 2.81 |
| Pulmonary embolism | 0, 0.00 | 1, 0.95 | 0, 0.00 | 1, 1.27 | 0, 0.00 | 0, 0.00 | 0, 0.00 | 2, 1.12 |
| Cardiac arrhythmia | 0, 0.00 | 1, 0.95 | 1, 0.97 | 5, 6.33 | 4, 7.14 | 1, 3.70 | 0, 0.00 | 6, 3.37 |
| Cysta renis | 2, 2.15 | 7, 6.67 | 11, 10.68 | 12, 15.19 | 11, 19.64 | 5, 18.52 | 2, 20.00 | 24, 13.48 |
| Nephrolithiasis | 2, 2.15 | 4, 3.81 | 5, 4.85 | 5, 6.33 | 4, 7.14 | 1, 3.70 | 0, 0.00 | 12, 6.74 |
| Varicocele | 1, 1.08 | 0, 0.00 | 0, 0.00 | 0, 0.00 | 0, 0.00 | 0, 0.00 | 0, 0.00 | 1, 0.56 |
| BPH | 0, 0.00 | 0, 0.00 | 3, 2.91 | 3, 3.80 | 7, 12.50 | 2, 7.41 | 0, 0.00 | 9, 5.06 |
| PCOS | 2, 2.15 | 2, 1.90 | 1, 0.97 | 0, 0.00 | 0, 0.00 | 0, 0.00 | 0, 0.00 | 2, 1.12 |
| Ovarian cysts | 3, 3.23 | 6, 5.71 | 9, 8.74 | 8, 10.13 | 6, 10.71 | 2, 7.41 | 0, 0.00 | 16, 8.99 |
| Glomerulonephritis | 0, 0.00 | 0, 0.00 | 0, 0.00 | 0, 0.00 | 0, 0.00 | 0, 0.00 | 0, 0.00 | 0, 0.00 |
| Seizures | 1, 1.08 | 2, 1.90 | 2, 1.94 | 1, 1.27 | 1, 1.79 | 0, 0.00 | 0, 0.00 | 3, 1.69 |
| Headache | 36, 38.71 | 45, 42.86 | 47, 45.63 | 37, 46.84 | 28, 50.00 | 11, 40.74 | 4, 40.00 | 103, 57.87 |
| Degenerative neurological disorder | 0, 0.00 | 0, 0.00 | 0, 0.00 | 0, 0.00 | 0, 0.00 | 0, 0.00 | 0, 0.00 | 0, 0.00 |
| Insomnia | 0, 0.00 | 1, 0.95 | 2, 1.94 | 4, 5.06 | 3, 5.36 | 1, 3.70 | 0, 0.00 | 6, 3.37 |
| Allergies | 21, 22.58 | 21, 20.00 | 18, 17.48 | 15, 18.99 | 12, 21.43 | 8, 29.63 | 0, 0.00 | 40, 22.47 |
| Autoimmune diseases | 4, 4.30 | 3, 2.86 | 3, 2.91 | 3, 3.80 | 3, 5.36 | 4, 14.81 | 2, 20.00 | 10, 5.62 |
| Dermatological conditions | 2, 2.15 | 1, 0.95 | 3, 2.91 | 2, 2.53 | 2, 3.57 | 3, 11.11 | 1, 10.00 | 6, 3.37 |
| Thyroid diseases | 3, 3.23 | 6, 5.71 | 8, 7.77 | 9, 11.39 | 6, 10.71 | 1, 3.70 | 0, 0.00 | 15, 8.43 |
| Kidney diseases | 4, 4.30 | 9, 8.57 | 13, 12.62 | 14, 17.72 | 12, 21.43 | 5, 18.52 | 2, 20.00 | 30, 16.85 |

**Table I**: Prevalence of comorbidities in the HAE patient population. Each cell shows the number of affected individuals and the percentage distribution of the corresponding disease in the corresponding age group.

Abbreviations: BPH=Benign prostatic hyperplasia; COPD=Chronic obstructive pulmonary disease; GERD=gastroesophageal reflux disease; IBD=Inflammatory bowel disease; PCOS=Polycystic ovary syndrome; PTX=Pneumothorax

**Table II:** Comorbidities in the HAE patient population versus the Hungarian general population as a whole

| **Comorbidity** | **HAE population (%)** | **Average Hungarian population (%)** | **ratio** |
| --- | --- | --- | --- |
| GERD | 20.79 | 20 | 1.04 |
| *Helicobacter pylori* seropositivity | 58.8 | 63.3 | 0.93 |
| Lactose intolerance | 4.49 | 39 | **0.12** |
| Celiac disease | 2.81 | 1.5 | 1.87 |
| Hemorrhoids | 4.49 | 10 | **0.45** |
| IBD | 1.12 | 0.5 | 2.24 |
| Heart failure | 4.49 | 1.6 | 2.81 |
| BPH | 5.06 | 2.7 | 1.87 |
| PCOS | 1.12 | 0.4 | 2.80 |
| Epilepsy | 1.69 | 0.3 | 5.63 |
| Insomnia | 3.37 | 9 | **0.37** |
| Eczema | 1.69 | 5 | **0.34** |

Bolded values indicate statistical significance.

Abbreviations: BPH=Benign prostatic hyperplasia; GERD=Gastroesophageal reflux disease; IBD=Inflammatory bowel disease; PCOS=Polycystic ovary syndrome

| **Comorbidity** | **Definition/Diagnostic criteria** |
| --- | --- |
| GERD | ICD-11 code DA22. Diagnosed by the gastroenterologist based on clinical symptoms, endoscopy reports |
| *Helicobacter pylori* infection | ICD-11 code DA60, DA63. Confirmed by biopsy, urea breath test, and stool antigen test reports |
| Lactose intolerance | ICD-11 5C61.6. Diagnosed based on clinical history, lactose tolerance test, hydrogen breath test |
| Cholecystolithiasis | ICD-11 DC11.3. Diagnosis based on abdominal ultrasound or imaging reports |
| Stomach ulcer | ICD-11 DA60.Y. Diagnosed by endoscopy or histological biopsy |
| Intussusception | ICD-11 DA91.0. Diagnosed by imaging (ultrasound, CT scan) |
| Celiac disease | ICD-11 DA95. Confirmed by serology (anti-tTG, anti-ema, anti- gliadin) and small bowel biopsy |
| Polyp of gallbladder | ICD-11 DC10.3 Diagnosed by ultrasound |
| Hemorrhoids | ICD-11 DB60 Clinical diagnosis by proctology or gastroenterology specialist reports |
| Liver disorders | ICD-11 DB9Z, DB92.Z (hepatic steatosis). Diagnosed based on ultrasound and some cases via histopathology. |
| IBD | ICD-11 DD70-72. Diagnosed by colonoscopy, biopsy, and clinical history in the gastroenterology department. |
| Acute pancreatitis | ICD-11 CD31. Diagnosed by clinical presentation, elevated enzymes, imaging (CT, ultrasound) in line with the Revised Atlanta Criteria. |
| Neoplasms | Histological confirmation from oncological documentation |
| Spondylosis | ICD-11 FA81. Diagnosis based on radiological documentation and additional rheumatological reports |
| Arthrosis | ICD-11 FA00-FA03. Diagnosis based on radiological documentation and additional rheumatological reports |
| Osteoporosis | ICD-11 FB83.1. Diagnosis based on dual energy x-ray absorptiometry (DEXA) scans and rheumatological reports |
| Diabetes | ICD-11 5A14, 5A11, 5A10. Diagnosis based on endocrinology reports, HbA1c, fasting glucose values |
| Hypothyroidism | ICD-11 5A00, 5A03.2. Diagnosed based on thyroid hormone tests, endocrinological documentation |
| Goiter | ICD-11 5A01. Diagnosed by clinical exam, ultrasound, and endocrinology reports |
| Thyroiditis | ICD-11 5A03. Diagnosed by clinical history, antibody tests, and endocrinology reports |
| Hyperthyroidism | ICD-11 5A02. Diagnosed based on thyroid function tests, ultrasound, and endocrinology reports |
| COPD | ICD-11 CA22. Diagnosis based on pulmonology reports |
| PTX | ICD-11 CB21. Diagnosed by chest X-ray or CT scan |
| Asthma bronchiale | ICD-11 CA23. Diagnosis based on pulmonology reports |
| Anxiety/depression | ICD-11 MB24.3, 6A71. Diagnosis based on psychiatry reports |
| Hyperlipidaemia | ICD-11 5C80.2 Diagnosis was based on laboratory values measured at our clinic (elevated triglyceride level was a criterion)  - Triglycerides: ≥ 1.7 mmol/L AND  - Total cholesterol: ≥ 5.2 mmol/L AND/OR - LDL-C: ≥ 3.3 mmol/L AND/OR - HDL-C: < 1.0 mmol/L (low)  Diagnosis was based on at least two lipid profiles on separate occasions to confirm persistent abnormality |
| Hypercholesterinaemia | ICD-11 5C80.0. Diagnosis was based on laboratory values measured at our clinic:  - Total cholesterol: ≥ 5.2 mmol/L AND/OR  - LDL-C: ≥ 3.3 mmol/L |
| Coronary artery disease | ICD-11 BA8Z,Y, BA52. Diagnosis based on cardiology reports, ECG, stress tests, angiography |
| Myocardial infarction | ICD-11 BA41. Diagnosed by clinical history, ECG, cardiac enzymes either at our clinic or based on cardiology reports |
| Hypertension | ICD-11 BA00. Diagnosed by repeated blood pressure measurements documented at our clinic (At least 2 separate occasions with elevated blood pressure readings (≥140/90 mmHg) or by the patient’s primary care center/general practitioner. |
| Heart failure | ICD-11 BD1Z. Diagnosed by cardiological evaluation, echocardiography, clinical records |
| Thrombosis | ICD-11 BD71.4. Diagnosed by imaging (ultrasound, CT angiography) and clinical records |
| Stroke | ICD-11 8B11. Diagnosed by neurological assessment and brain imaging (neurological reports) |
| Pulmonary embolism | ICD-11 BB00 Diagnosed by CT pulmonary angiography, ventilation-perfusion scans |
| Cardiac arrhythmia | ICD-11 BC9Y Diagnosed by ECG and cardiology reports |
| Cysta renis | ICD-11 GB80.0, GB80.1 Diagnosed by ultrasound at our clinic. |
| Nephrolithiasis | ICD-11 GB70.0. Diagnosed by imaging (ultrasound, CT) and urology/nephrology reports |
| Varicocele | ICD-11 BD75.1. Diagnosed by clinical examination and scrotal ultrasound from urology reports |
| BPH | ICD-11 GA90. Diagnosed by urology reports |
| PCOS | ICD-11 5A80.1. Diagnosed by endocrinology reports, ultrasound, and clinical criteria (Rotterdam). |
| Ovarian cysts | ICD-11 GA18.6. Diagnosed by pelvic ultrasound |
| Glomerulonephritis | ICD-11 GB40. Diagnosed by nephrology reports, urinalysis, biopsy |
| Seizures | ICD-11 8A68. Diagnosis based on neurological reports |
| Headache | ICD-11 Based on patient disclosure on each follow-up at our center with a comprehensive evaluation of headache classification and specific features potentially associated with HAE. |
| Degenerative neurological disorders | ICD-11 6D81-85. Diagnosis based on neurological reports |
| Insomnia | ICD-11 7A00-01. Diagnosed by clinical history and sleep specialist evaluation |
| Allergies | Based on clinical history and at least one objective evidence of sensitization: skin prick testing/patch testing, serum specific IgE testing (allergen-specific IgE antibody measurements), total IgE, challenge tests or allergology reports based on these parameters. |
| Autoimmune diseases | Diagnosis is confirmed by an immunologist at our center or obtained from medical reports. Diagnoses are based on a combination of clinical presentation, positive autoantibodies (e.g., ANA, anti-dsDNA, etc…), inflammatory markers, imaging and/or biopsy results, depending on the specific condition. |
| Dermatological conditions | Diagnoses based on dermatological documentation (clinical evaluation, dermatologic examination, and, when applicable, histopathological confirmation) |
| Thyroid diseases | Diagnoses based on endocrinological documentation |
| Kidney diseases | Diagnoses based on nephrological documentation |

**Table III**: Comorbidities with corresponding definitions and diagnostic criteria

Abbreviations: BPH=Benign prostatic hyperplasia; COPD=Chronic obstructive pulmonary disease; GERD=gastroesophageal reflux disease; IBD=Inflammatory bowel disease; ICD= International Classification of Diseases; PCOS=Polycystic ovary syndrome; PTX=Pneumothorax
